# Supplementary material for: Vaeda computationally annotates doublets in single-cell RNA sequencing data
Source: Bioinformatics. 2022 Nov 7;39(1):btac720. doi: 10.1093/bioinformatics/btac720 (PMC9805559; doi:10.1093/bioinformatics/btac720)
Supplement: btac720_Supplementary_Data [file btac720_supplementary_data.pdf]

Supplementary Information

For the manuscript

Vaeda computationally annotates doublets in single-cell RNA sequencing data

Hannah Schriever [1,2] and Dennis Kostka [1,3,*]

[1]Department of Developmental Biology, University of Pittsburgh, Pittsburgh, PA, 15201, USA and
[2]Canegie Mellon – University of Pittsburgh Joint PhD Program, University of Pittsburgh, Pittsburgh, PA, 15201, USA and
[3]Department of Computational & Systems Biology and Center for Evolutionary Biology and Medicine, University of Pittsburgh, Pittsburgh, PA, 1501, USA.
[*]To whom correspondence should be addressed.

S1 Supplemental Figures

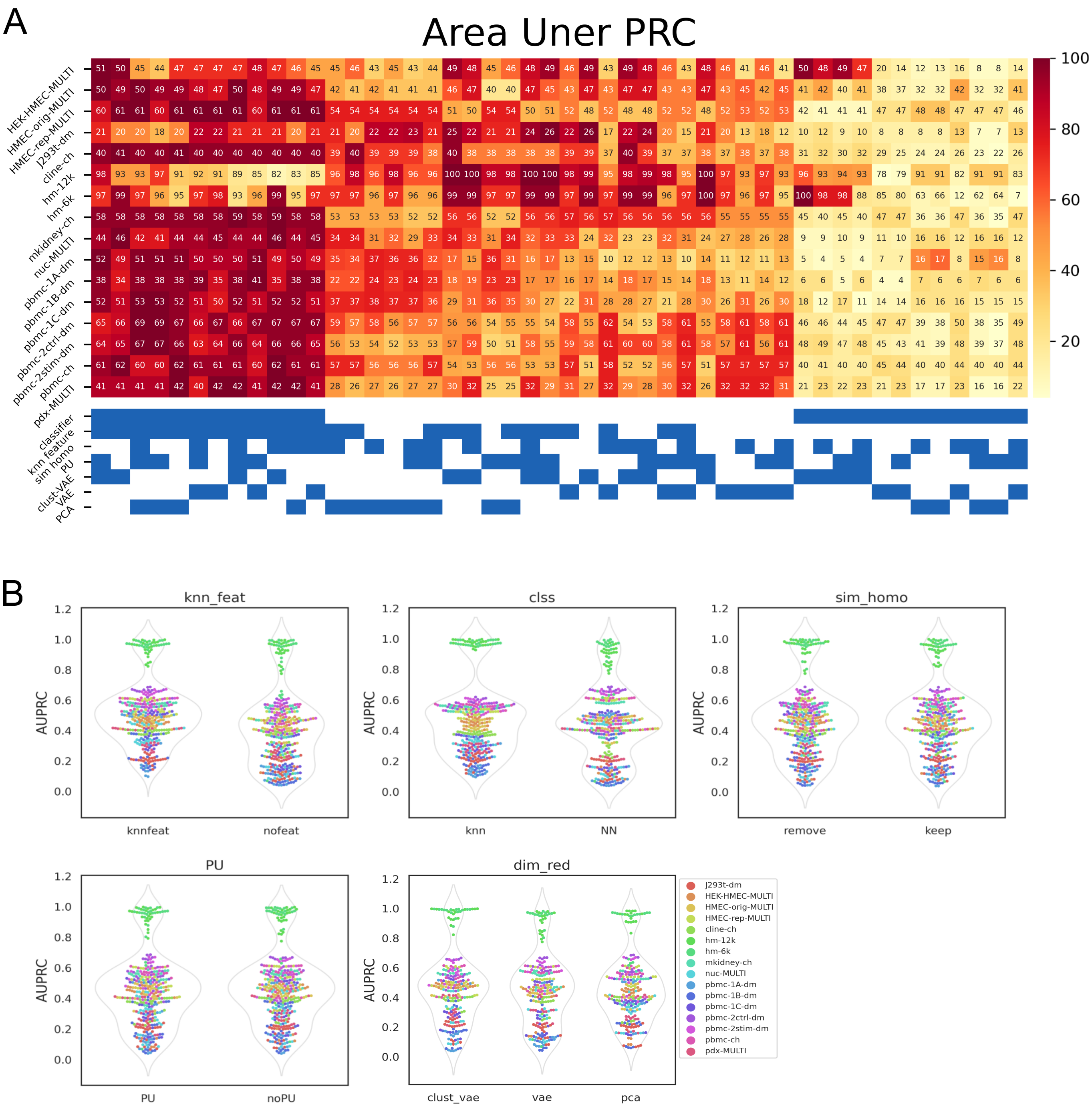

Supplemental Figure S1: ***Ablation Analysis shows the importance of combining knn and neural network classifier.*** (A) Heat map showing area under the precision recall curve for every permutation on the components of vaeda (columns) on each benchmarking dataset (rows). Colors are normalized by row and correspond to performance with burgundy being the best and pale yellow being the worst. Bottom chart depicts which components are used. The key is as follows. classifier: blue=neural network classifier, white=knn classifier; knn_feature: blue=append to low dimensional representation, white=no knn_feature; sim_homo: blue=include simulated homotypic doublets, white=remove simulated homotypic doublets; PU: blue=PU loop implemented, white=PU not used. Then for clust_vae, vae, and PCA blue indicates that methods was used for dimensionality reduction and white indicates it was not. (B) Violin plots showing AUPRC for every permutation on the components of vaeda, with each plot focusing on the significance of one component.

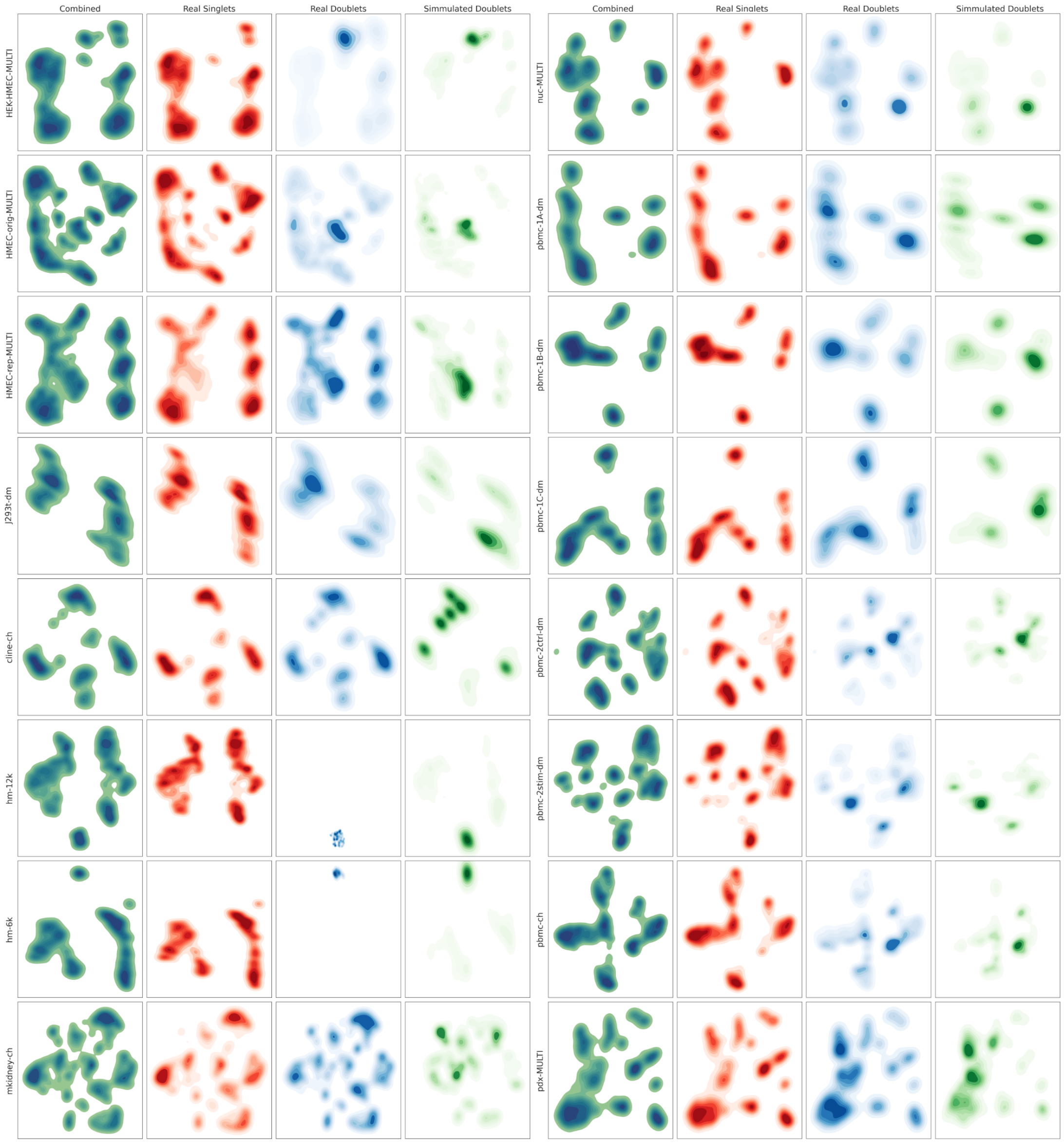

Supplemental Figure S2: ***Simulated doubles largely agree with experimentally annotated doublets.*** Shown are two-dimensional projections of vaeda's latent representation of cells. Rows are different benchmark datasets. The first column in each panel are all cells in the dataset; the second and third columns show experimentally annotated singlets and doublets, respectively; the third column shows vaeda's simulated doublets.

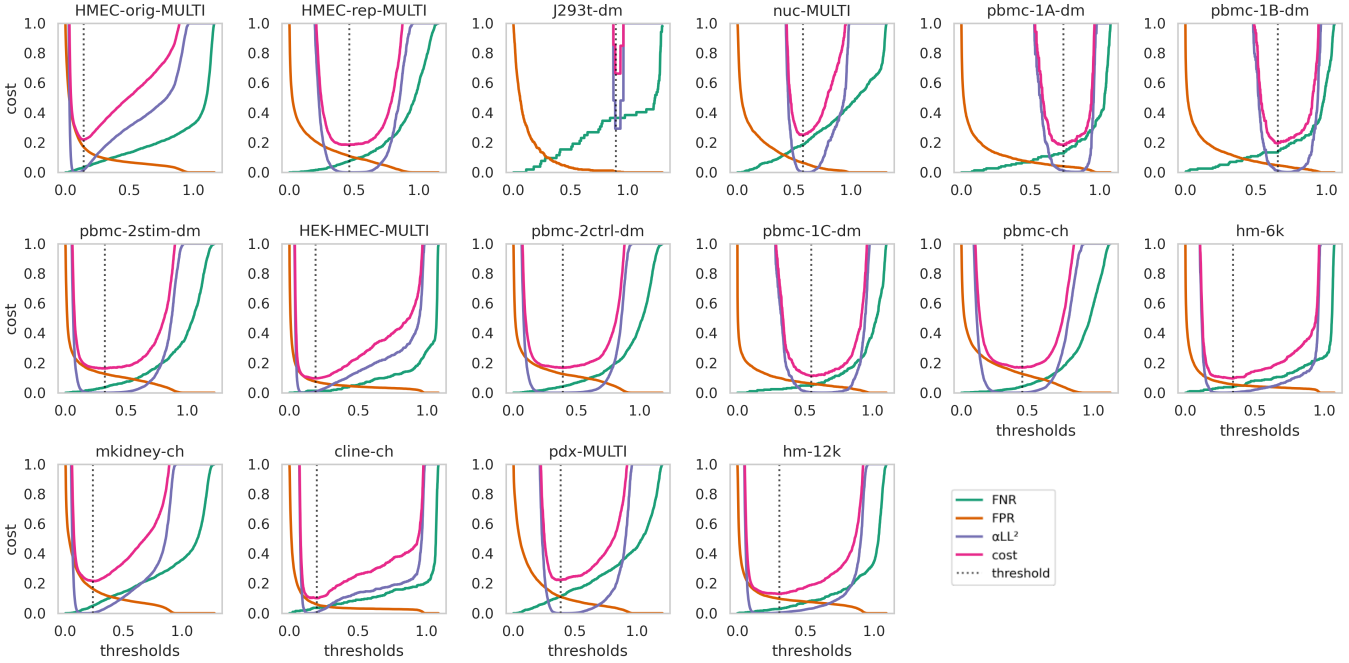

Supplemental Figure S3: ***Cost functions used for thresholding during doublet calling.*** Depicted above are the cost functions, along with the components of the cost functions for each dataset. FNR=false negative rate; FPR=false positive rate; LL^2=likelyhood squared; cost=FNR+FPR+α LL^2; threshold=minimum of the cost function.

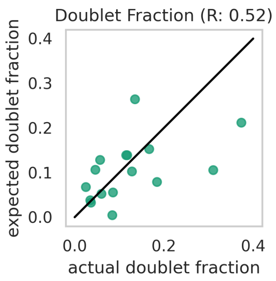

Supplemental Figure S4: ***Expected number of doublets vs actual number of doublets.*** Each point represents one of the benchmarking datasets. Expected doublet fraction correlates with actual doublet fraction with a correlation coefficient of 0.52.

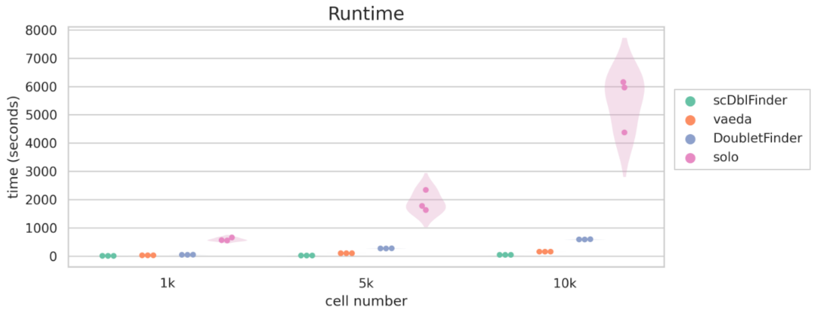

Supplemental Figure S5: ***Running time.*** Running time of scDblFinder, vaeda, DoubletFinder, and solo in seconds.

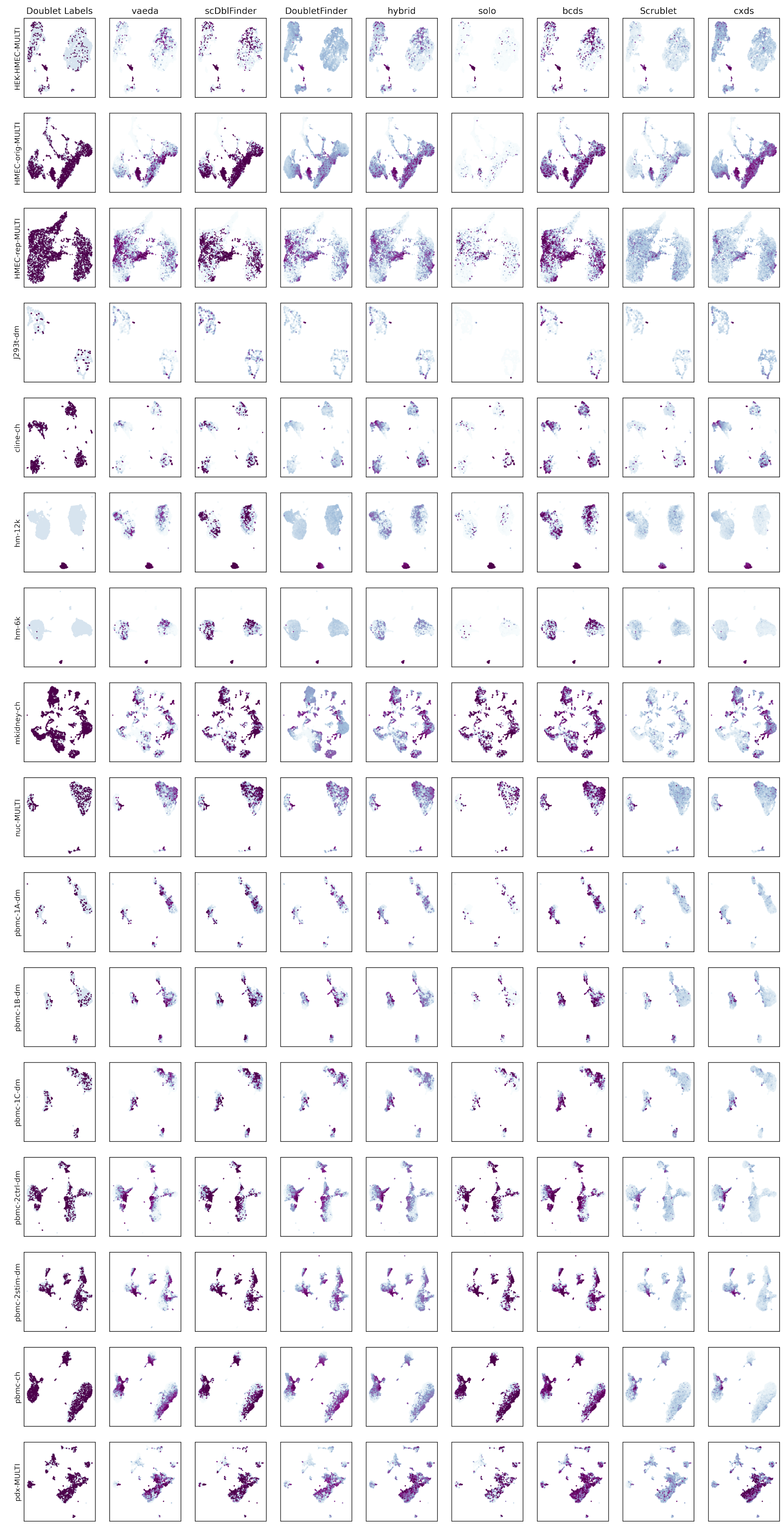

Supplemental Figure S6: ***Score distributions from each method projected onto UMAP representations of each benchmarking dataset.*** Depicted above are UMAP projections on the top 30 Principal Components of each dataset. Rows are datasets, the first column is the experimental doublet annotations, and the remaining columns each are a method. In the first column, purple=doublet; pale blue=singlet. In the remaining columns, dark purple represents higher doublet scores while pale blue represents lower scores.

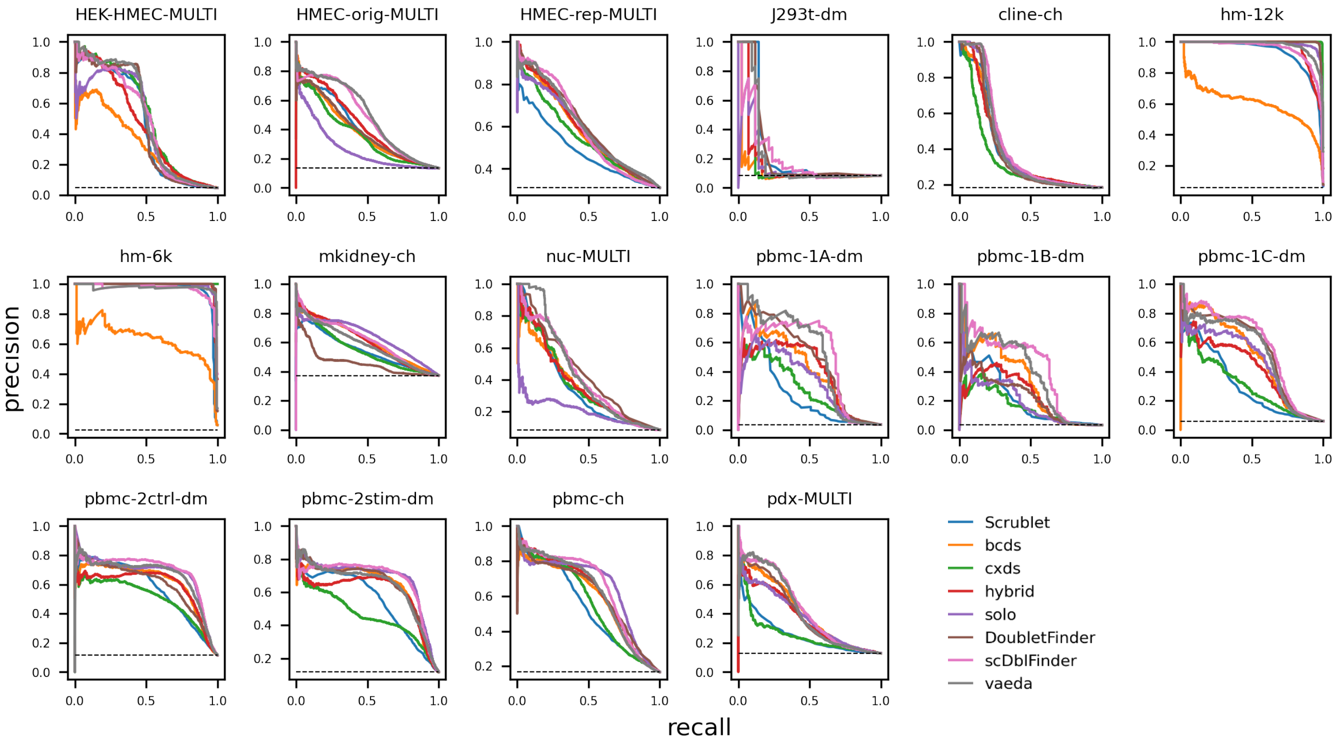

Supplemental Figure S7: ***Precision Recall Curves for each method.*** Shown above are the Precision Recall Curves for each method applied to each dataset.

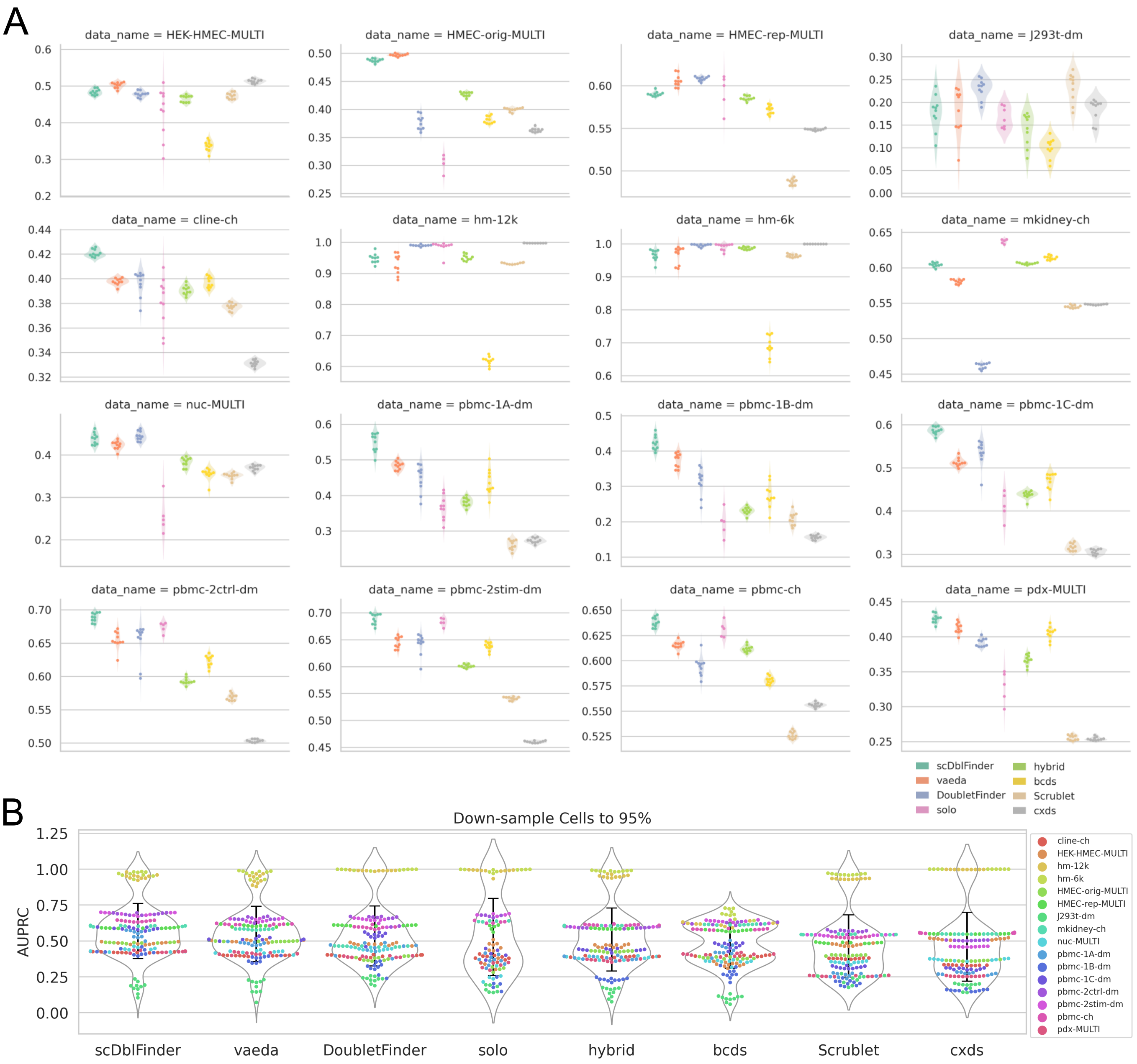

C

method	wins	ties	losses
**scDblFinder**	83	13	16
**vaeda**	67	17	28
**DoubletFinder**	64	16	32
**solo**	46	23	43
**hybrid**	43	13	56
**bcds**	41	8	63
**Scrublet**	20	10	82
**cxds**	32	4	76

Supplemental Figure S8: ***Area under the Precision Recall Curve for methods applied to down-samplings of each dataset.*** (A) Violin plots depicting area under the Precision Recall Curve for each down-sampling across methods and datasets. (B) Violin plots of AUPRC for each method on 95% down-sampled datasets. (C) Table showing all pairwise comparisons of doublet detection methods stratified by dataset, using paired Wilcoxon rank-sum tests to decide "wins" ($p \leq 0.05$, higher performance), "ties" ($p > 0.05$), and "losses" ($p \leq 0.05$, lower performance)

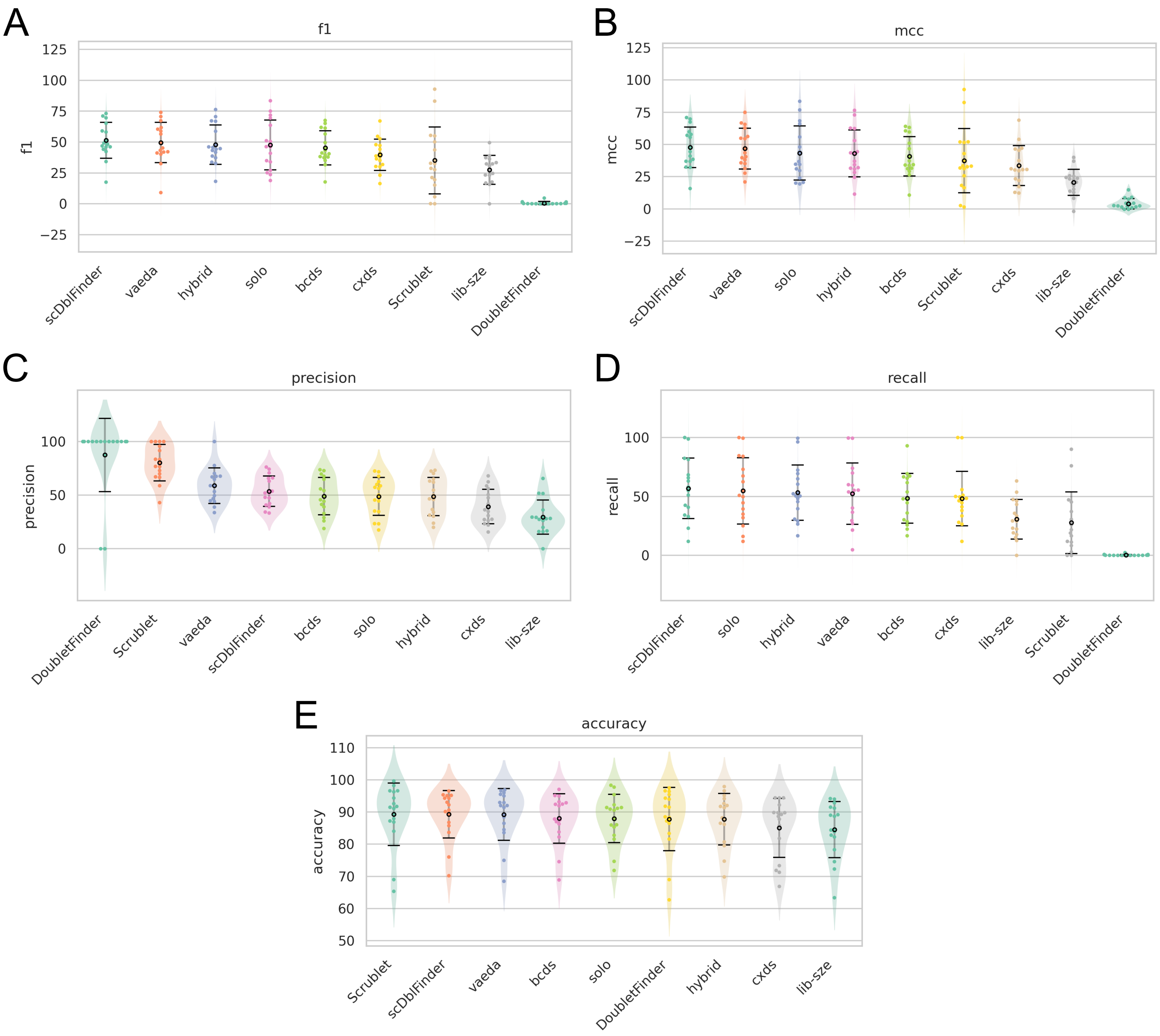

Supplemental Figure S9: ***Doublet Calling performance varies highly with dataset.*** Shown are violin plots of metrics (f1 (A), mcc(B), precision(C), recall(D), and accuracy (E)) for accessing doublet calling. Methods are given on the x axis and ordered by decreasing performance. Each point represents one of the benchmarking datasets and the black point is the mean. Error bars represent the variance.

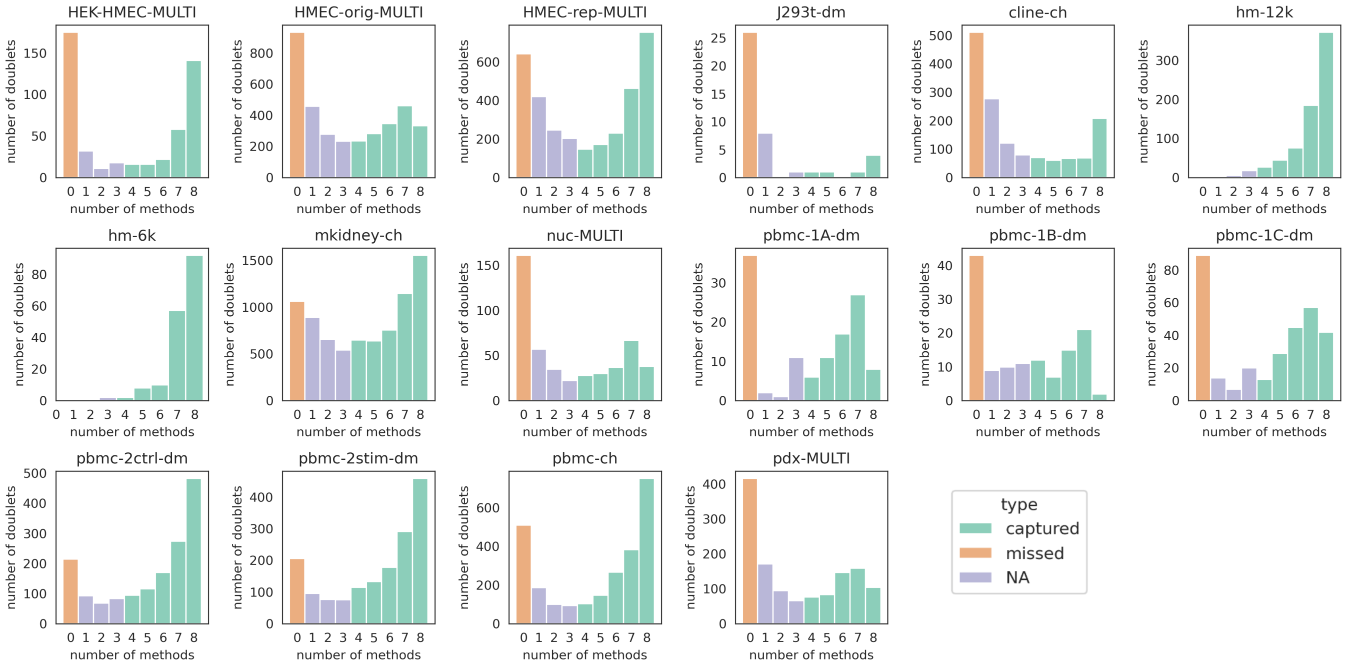

Supplemental Figure S10: ***Most datasets have a sizable population of doublets that are misclassified by every method.*** Depicted above are histograms of the number of doublets that are classified by 0, 1, 2, 3, 4, 5, 6, or 8 of the methods. We define ”missed” doublets as doublets that are classified by none of the methods and ”captured” doublets as doublets that are classified by at least 4 of the methods.

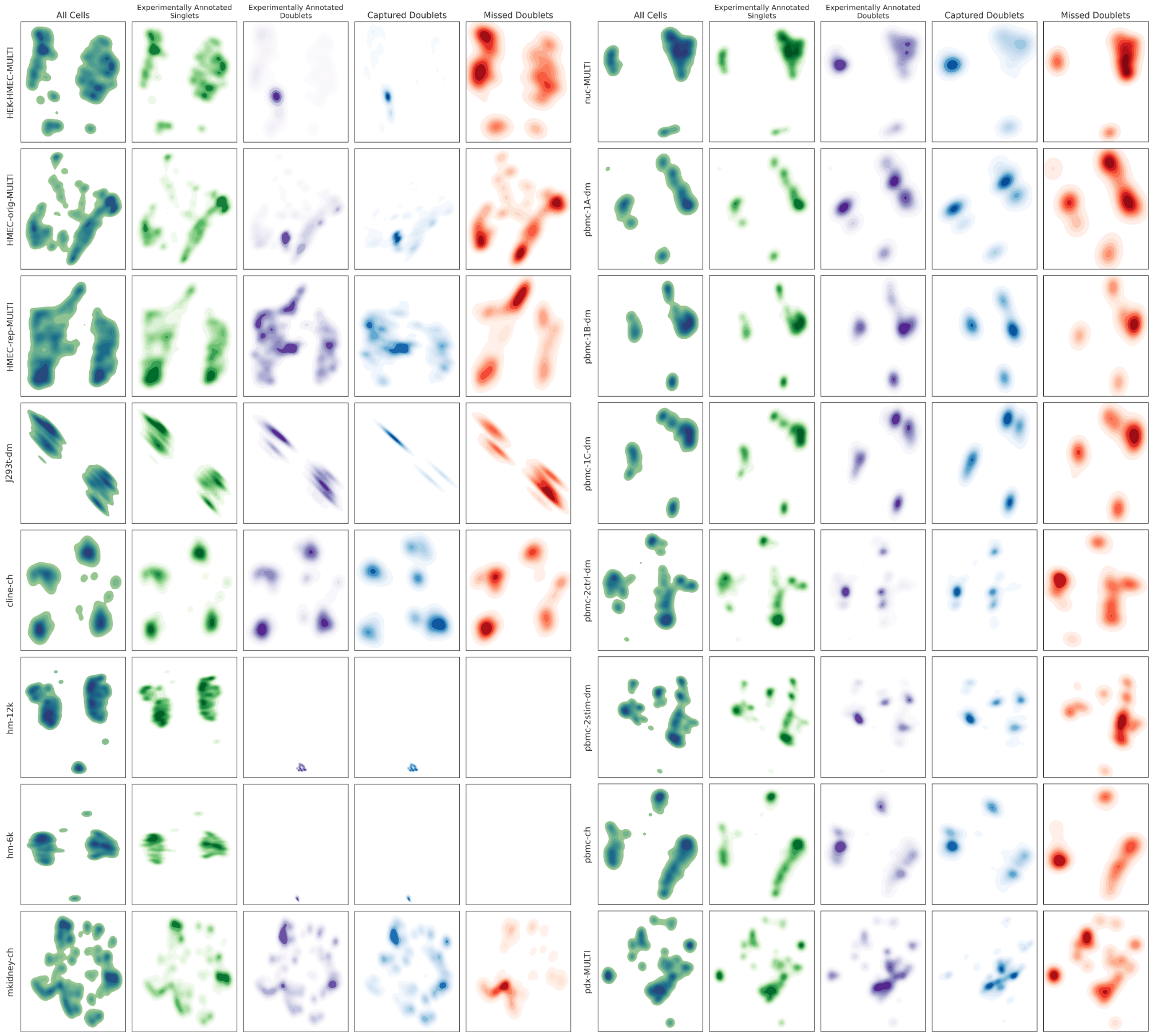

Supplemental Figure S11: ***Doublets misclassified by all methods have greater mixing with singlets.*** Shown are UMAP projections of top 30 principal components of each dataset. Rows are the benchmarking datasets. The first column in each panel are all cells in the dataset; the second and third columns show experimentally annotated singlets and doublets, respectively; the third column shoes ”captured” doublets, and the fourth column shows ”missed” doublets. Where ”captured” doublets are experimentally annotated doublets classified by at least 4 of the 8 methods and ”missed” doublets are experimentally annotated doublets that are misclassified by all of the methods.

S2 Supplemental Tables

dataset	# of cells	# of doublets	% doublets	median UMI	median gene count	annotation method
HEK-HMEC-MULTI	10,641	489	4.6	17,424	3,795	MULTI-seq
HMEC-orig-MULTI	26,426	3,568	13.5	23,502	4,598	MULTI-seq
HMEC-rep-MULTI	10,580	3,282	31.0	1,188	601	MULTI-seq
J293t-dm	500	42	8.4	14,135	3,461	demuxlet
cline-ch	7,954	1,465	18.4	4,825	2,149	cell hashing
hm-12k	12,820	730	5.7	12,424	3,147	species mixture
hm-6k	6,806	171	2.5	21,301	4,032	species mixture
mkidney-ch	21,179	7,901	37.3	3,929	1,687	cell hashing
nuc-MULTI	5,578	475	8.5	1,021	786	MULTI-seq
pbmc-1A-dm	3,298	120	3.6	973	384	demuxlet
pbmc-1B-dm	3,790	130	3.4	862	361	demuxlet
pbmc-1C-dm	5,270	316	6.0	829	352	demuxlet
pbmc-2ctrl-dm	13,913	1,598	11.5	1,276	526	demuxlet
pbmc-2stim-dm	13,916	1,631	11.7	1,361	550	demuxlet
pbmc-ch	15,272	2,545	16.7	556	323	cell hashing
pdx-MULTI	10,296	1,317	12.8	2,243	1,029	MULTI-seq

Supplemental Table S1: Benchmarking datasets.

	vaeda-scDblFinder	vaeda-DoubletFinder	scDblFinder-DoubletFinder
HEK-HMEC-MULTI	vaeda	vaeda	≈
HMEC-orig-MULTI	vaeda	vaeda	scDblFinder
HMEC-rep-MULTI	vaeda	≈	DoubletFinder
J293t-dm	≈	DoubletFinder	DoubletFinder
cline-ch	scDblFinder	≈	scDblFinder
hm-12k	≈	DoubletFinder	DoubletFinder
hm-6k	≈	DoubletFinder	DoubletFinder
mkidney-ch	scDblFinder	vaeda	scDblFinder
nuc-MULTI	scDblFinder	DoubletFinder	≈
pbmc-1A-dm	scDblFinder	vaeda	scDblFinder
pbmc-1B-dm	scDblFinder	vaeda	scDblFinder
pbmc-1C-dm	scDblFinder	DoubletFinder	scDblFinder
pbmc-2ctrl-dm	scDblFinder	≈	scDblFinder
pbmc-2stim-dm	scDblFinder	≈	scDblFinder
pbmc-ch	scDblFinder	vaeda	scDblFinder
pdx-MULTI	scDblFinder	vaeda	scDblFinder

Supplemental Table S2: *Comparison of top three methods stratified by dataset.* Wilcoxon rank-sum tests were used to identify significant performance (p¡=0.05) differences between the top three methods on the 95% down-samplings. $\approx$ implies $p > 0.05$.

	f1	mcc	precision	recall	accuracy
scDblFinder	50.1	46.9	55.5	55.6	88.8
vaeda	48.5	45.3	56.4	53.9	88.3
hybrid	45.9	42.6	53.8	51.4	88.0
solo	44.9	41.1	49.9	50.3	88.0
bcds	45.3	41.4	50.4	50.5	87.9
cxds	39.0	34.6	46.2	44.7	86.5
scrublet	38.8	34.4	45.9	44.3	86.5
lib-sze	27.5	20.6	29.5	30.7	84.5
DoubletFinder	45.1	41.3	52.9	50.3	87.5

Supplemental Table S3: Doublet classification across 16 benchmark datasets (averaged performance). Instead of using each method's doublet callers, we took the top $n^2 * 10^{-5}$ scoring doublets.

	f1	mcc	precision	recall	accuracy
scDblFinder	1.3	0.9	-1.8	1.3	0.5
vaeda	1.2	1.4	2.5	-1.4	0.9
hybrid	1.9	0.4	-5.0	1.9	-0.3
solo	2.7	2.2	-1.1	4.5	-0.1
bcds	-0.1	-0.5	-1.4	-1.9	0.1
cxds	0.6	-0.9	-6.9	3.6	-1.4
scrublet	-3.6	3.1	34.4	-16.6	2.8
lib-sze	0.0	0.0	0.0	0.0	0.0
DoubletFinder	-44.4	-37.2	0.346	-50.0	0.3

Supplemental Table S4: Difference between doublet caller metrics and expected doublet classification metrics (negative means using expected number of doublets performs better than the methods built in doublet caller)

	f1	mcc	precision	recall	accuracy
scDblFinder	57.7	50.7	57.7	57.7	88.9
vaeda	56.1	48.8	56.1	56.1	88.6
DoubletFinder	53.8	45.9	53.8	53.8	87.8
hybrid	53.1	45.7	53.1	53.1	88.2
solo	52.9	45.6	52.9	52.9	88.4
bcds	48.9	41.3	48.9	48.9	87.8
cxds	47.2	38.8	47.2	47.2	86.7
Scrublet	46.4	38.0	46.4	46.4	86.7
lib-sze	29.2	19.4	29.2	29.2	84.3

Supplemental Table S5: Doublet classification across 16 benchmark datasets (averaged performance). Instead of using each method's doublet callers, we set the number of called doublets to the number of annotated doublets
